# *Aspergillus caespitosus* ASEF14, an oleaginous fungus as a potential candidate for biodiesel production using sago processing wastewater (SWW)

**DOI:** 10.1186/s12934-021-01667-3

**Published:** 2021-09-09

**Authors:** Naganandhini Srinivasan, Kiruthika Thangavelu, Ashika Sekar, B. Sanjeev, Sivakumar Uthandi

**Affiliations:** 1 Biocatalysts Laboratory, Deptartment of Agricultural Microbiology, Tamil Nadu Agricultural University, Coimbatore, 641 003 India; 2 Department of Renewable Energy Engineering, Agricultural Engineering College & Research Institute, Tamil Nadu Agricultural University, Coimbatore, 641 003 India

**Keywords:** *Aspergillus*, Biodiesel, Lipid content, Sago wastewater, Decontamination

## Abstract

**Background:**

Oleaginous microorganisms are sustainable alternatives for the production of biodiesel. Among them, oleaginous fungi are known for their rapid growth, short life cycles, no light requirement, easy scalability, and the ability to grow in cheap organic resources. Among all the sources used for biodiesel production, industrial wastewater streams have been least explored. We used oleaginous fungi to decontaminate sago processing wastewater and produce biodiesel.

**Results:**

Among the 15 isolates screened for lipid production and starch utilization using the Nile red staining assay and amylase plate screening, three isolates accumulated > 20% (w/w) of their dry cell mass as lipids. The isolate ASEF14 exhibited the highest lipid accumulation (> 40%) and was identified as *Aspergillus caespitosus* based on the 28S rRNA gene sequencing. The maximum lipid content of 54.4% in synthetic medium (SM) and 37.2% in sago processing wastewater (SWW) was produced by the strain. The Fourier-transform infrared (FTIR) spectroscopy of the fungal oil revealed the presence of functional peaks corresponding to major lipids. Principal component analysis (PCA) of the FTIR data revealed major changes in the fatty acid composition during the transition from the growth phase (Days 1–3) to the lipid accumulation phase (Days 4–7). The fatty acid methyl esters (FAME) analysis of fungal oil from SWW contained 43.82% and 9.62% of saturated and monounsaturated fatty acids, respectively. The composition and percentage of individual FAME derived from SWW were different from SM, indicating the effect of nutrient and fermentation time. The fuel attributes of the SM- and SWW-grown fungal biodiesel (kinematic viscosity, iodine value, cetane number, cloud and pour point, linolenic acid content, FA > 4 double bonds) met international (ASTM D6751, EN 14214) and national (IS 15607) biodiesel standards. In addition to biodiesel production, the strain removed various contaminants such as total solids (TS), total suspended solids (TSS), total dissolved solids (TDS), dissolved oxygen (DO), chemical oxygen demand (COD), biological oxygen demand (BOD), total nitrogen (TN), total phosphorus (TP), and cyanide up to 58.6%, 53.0%, 35.2%, 94.5%, 89.3%, 91.3%, 74.0%, 47.0%, and 53.84%, respectively, from SWW.

**Conclusion:**

These findings suggested that *A. caespitosus* ASEF14 is a potential candidate with high lipid accumulating ability (37.27%), capable of using SWW as the primary growth medium. The medium and incubation time alter the FAME profile of this fungus. The physical properties of fungal oil were in accordance with the biodiesel standards. Moreover, it decontaminated SWW by reducing several polluting nutrients and toxicants. The fungal biodiesel produced by this cost-effective method could serve as an alternate path to meet global energy demand.

## Background

With the increasing depletion of fossil fuels, there is an urgent need to search for viable substitutes to fulfill the energy requirements of society. A suitable substitute for fossil fuels would be an alternative fuel with superior environmental benefits over fossil fuels, economically competitive, and producible in sufficient quantities to meet the increasing energy demands. In addition, it must provide a net energy gain over the energy sources used to produce it [1]. Recently, biodiesel has received considerable attention as an alternative to fossil fuels. Although more than 80% of biodiesel is obtained from plant oil globally, it is unable to meet the current demand. Furthermore, it creates a fuel versus food fight, with negative consequences on consumers and the agricultural system.

The most hindering aspect of the commercialization of biodiesel production has been the high cost of raw material, especially that of currently used plant oils [2]. The oily or oleaginous microbes are being investigated worldwide as an alternative sustainable feedstock to plant oils for biodiesel production [3]. These oil-rich microbes can accumulate fatty acids (FA) up to 70% of their dry weight, with a predominance of (mono)unsaturated fatty acids species, which is similar to plants, different from animals [4]. In addition, these microbes do not compete either with food, feed crops, or agricultural land and have the ability to manufacture and accumulate high quantities of triacylglycerols (TAGs). These TAGs could be converted to biodiesel through transesterification, which involves converting methanol and TAGs into biodiesel in the presence of an acid or base as a catalyst [5].

Oleaginous yeast and fungi have emerged as the most promising candidate for biodiesel production due to their optimal and diverse fatty acid profiles, pelleted growth that allows easier and cost-effective downstream processing, ability to degrade and grow on a wide range of renewable sources, such as lignocellulosic biomass [6–8], waste glycerol [9], waste cooking oil [10, 11], and industrial waste streams [12–14]. Industrial wastewater streams have been least explored.

The production of sago from tapioca is one of the most significant agro-based food industries in South-East Asia. There are approximately 1000 sago-processing factories that are currently operational in Salem and Namakkal Districts of Tamil Nadu, India. Sago industrial plants release a huge volume of wastewater rich in organic and inorganic compounds, especially starch. The waste is discharged into the nearby river, causing detrimental effects to the environment. Therefore, recycling, reprocessing, and reuse of these waste streams in an eco-friendly manner is important. All organic waste materials contain adequate quantities of nutrients that support the growth and metabolism of microbes. The use of waste streams as a substrate for oleaginous microbes would be an effective strategy to improve the process economics of biodiesel production [15, 16]. Earlier reports have suggested the potential of industrial waste streams for microbial lipid production using oleaginous microbes. For example, Xue et al. [12] successfully cultured the oleaginous yeast *Rhodotorula glutinis* in monosodium glutamate wastewater, producing 25 g L^–1^ biomass with 25% lipid content. Similarly, a culture of *Aspergillus niger* accumulated 41% to 57% of lipids in biodiesel-derived waste glycerol [9]. Muniraj et al. [13] used various diluted potato-processing wastewater (PPW) as a growth medium for *A. oryzae* and obtained a 3.5 g L^–1^ lipid yield at 25% diluted PPW. In addition, the removal of COD, total soluble nitrogen, and total soluble phosphorus up to 91%, 98%, and 97%, respectively, were achieved. *A. flavus* I16–3 and *M. rouxii* grew well and efficiently used the starch in PPW and produced 2.8 and 3.6 g L^–1^ of lipids, respectively [17].

We previously reported that the oleaginous yeast *Candida tropicalis* ASY2 produced 1.21 g L^–1^ of lipid (lipid content: 48.59%) when cultivated in SWW [14, 18]. Further, we characterized the yeast biomass for its bioenergy applications [19]. In the present study, we screened different morpho-type fungi from SWW for its amylase production and oleaginicity, and investigated the growth, lipid production and decontaminating ability of *A. caespitosus* ASEF14 in SWW. The fungal biomass generated was used to produce FAMEs by direct transesterification and evaluated for potential biodiesel’s fuel properties.

## Materials and methods

### Chemicals and reagents

Nile red (NR) dye and FAME mix was purchased from Sigma-Aldrich Pvt. Ltd. (St Louis, MO, USA). Analytical grade solvents, such as isopropanol, ethanol, chloroform, methanol, and other chemicals, were obtained from commercial suppliers such as HiMedia, Merck, Central Drug House, and SRL Laboratory.

### Sago-processing wastewater (SWW) collection and analysis

SWW was collected in an air-tight container from Sri Senthil Andavar Sago industry located in Salem District, Tamil Nadu, India, and stored at 4 °C. The physicochemical properties of SWW were analyzed immediately after transport, following the standard method of water and wastewater analysis [20]. Biological oxygen demand (BOD) was measured according to the closed bottle test, and chemical oxygen demand (COD) was measured by the dichromate method using the Soxhlet apparatus. The total nitrogen and phosphorus were determined by Kjeldahl [21] and colorimetric methods, respectively. The starch in SWW was analyzed using the phenol–sulfuric acid method [22]. Cyanide content in SWW was determined using a modified picric acid method [23].

### Biotrap enrichment and isolation

**Fig. 1:**
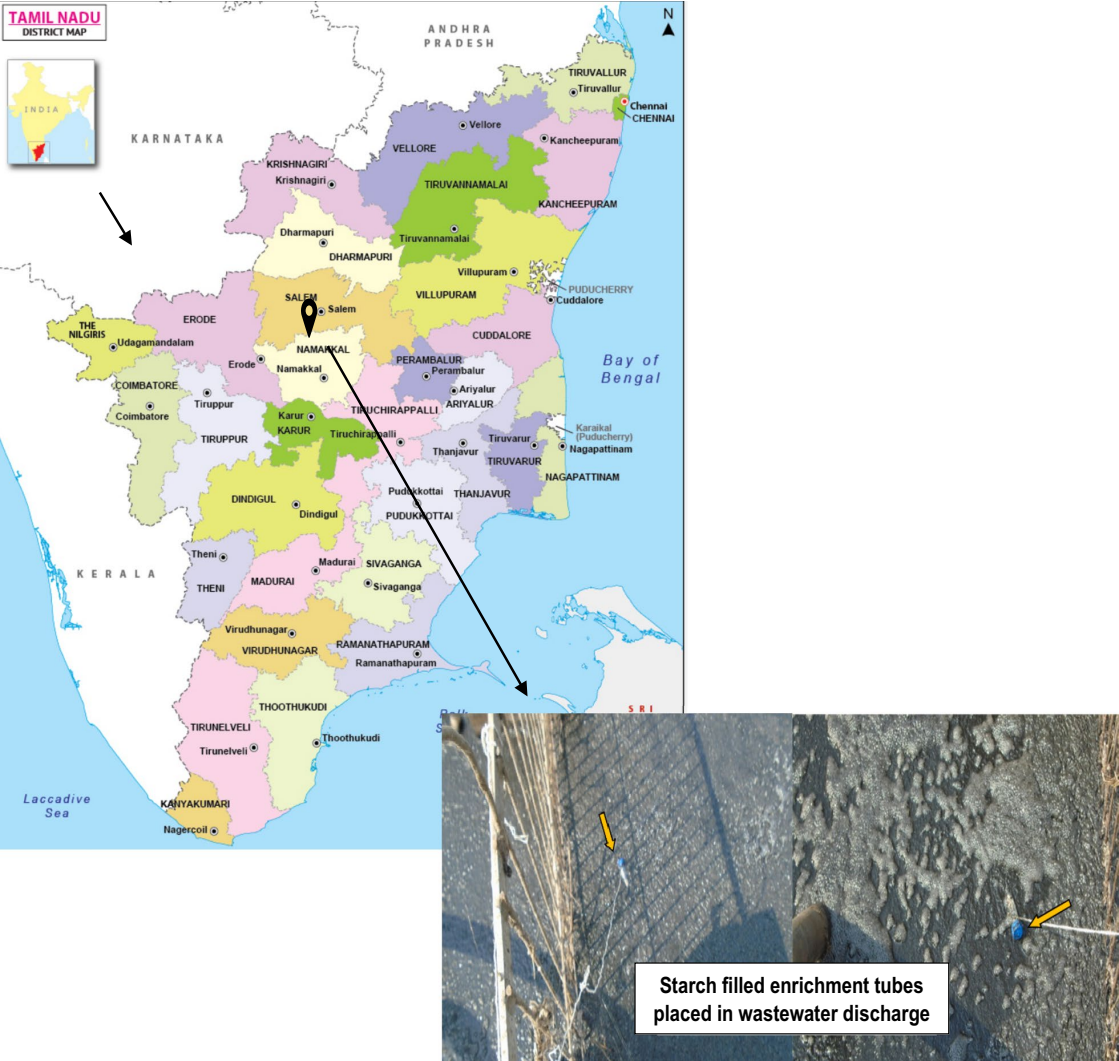
Enrichment and sample collection from sago-processing industries (location: Salem district, Tamil Nadu, India)

The biotrap enrichment approach was used to isolate oleaginous and starch hydrolyzing fungi from SWW. In this procedure, soluble starch (HiMedia, India) and a substrate called “thippi” (a fibrous waste fraction left in the siever after milky starch passed out during the starch extraction process) were mixed and filled to one-third portion of a 15 mL Falcon tube. The tube was closed and then placed in a wastewater discharge pipeline. A few micro-holes were made on the surface of the Falcon tube before it was placed in the discharge line in order to keep the tube content in contact with SWW. The enrichment process was carried out for 15 days, as recommended [24–26]. In this way, the starch-utilizing fungi in the SWW were selectively flourished in the tube (Fig. 1).

After 15 days, the enriched samples were serially diluted up to 10^−4^ fold dilution. An aliquot of 1 mL from the final dilutions was plated on sterile potato dextrose agar (PDA) medium containing ampicillin (0.1 mg mL^–1^) and incubated at room temperature (28 ± 2 °C) for 3 to 5 days. Single colonies of fungus were isolated, purified using either single spore or single hyphal tip method, and maintained on PDA slants at 4 °C.

### Screening of oleaginous fungi

#### Starch-hydrolyzing activity

Purified fungal isolates were screened for amylase activity by cultivating on starch agar medium containing the following chemical components per liter of the medium: starch, 1 g; peptone, 0.5 g; yeast extract, 0.3 g; NaCl, 0.3 g; and agar, 20 g [27]. Individual discs of each fungal isolate that had grown on the starch agar medium for 3 to 5 days were flooded with 1% iodine solution and observed for clear zones around the fungal colony.

#### Nile red staining

All amylase-positive isolates were evaluated for intracellular lipid bodies (LBs), indicative of lipid accumulation, by NR fluorescence staining [28, 29]. An aliquot of 10 µL of NR (0.1 mg mL^–1^) was mixed with the fungal culture suspension, followed by the addition of 2 mL of phosphate-buffered saline (PBS) to 50 µL of culture broth, which was subsequently maintained in the dark for 5 min. The sample was analyzed under a Nikon Eclipse 80i light microscope (Nikon Instruments, Tokyo, Japan) equipped with a digital camera, using a 465 to 495 nm excitation filter, a 505 nm diachronic mirror, and a 515 to 535 nm barrier filter.

### Lipid content determination

The selected fungal isolates were grown in 250 mL Erlenmeyer flasks containing 50 mL of modified starch-based SM (composition per liter: starch, 30 g; ammonium sulfate, 0.5 g; potassium dihydrogen phosphate, 7 g; disodium hydrogen phosphate, 2.5 g; magnesium sulfate, 1.5 g; ferric chloride, 0.15 g; calcium chloride, 0.15 g; zinc sulfate, 0.02 g; and manganese sulfate, 0.06 g) with a wide C:N ratio of 30:1 for seven days at 30 °C [30]. After incubation, the fungal biomass from the broth culture was separated by filtration, washed with sterile distilled water and oven-dried at 60 °C for 15 h [31]. Dried samples were used to determine the lipid content using the method described by Folch et al. [32], using chloroform and methanol (2:1) as solvents.

### Phylogeny and identification of ASEF14

Genomic DNA was extracted from the high lipid accumulating fungal isolate ASEF14 using the modified CTAB method [33]. DNA concentration and purity were analyzed by Nanodrop™ 2000 spectrophotometer (Thermo-Fisher, USA) and agarose gel electrophoresis, respectively. PCR amplification of 28S rRNA region was performed in a T-gradient thermocycler (BioRad T100 Thermal Cycler, USA) using the universal primers ITS1 (F) 5'-CTTGGTCATTTAGAGGAAGTAA-3’ and ITS4 (R) 5'-TCCTCCGCTTATTGATATGC-3’, under the following conditions: initial denaturation at 95 °C for 5 min, followed by 33 cycles of 94 °C for 30 s (denaturation), 58 °C for 1.5 min (annealing), 72 °C for 2.5 min (primer extension), and a final extension at 72 °C for 7 min. The amplified PCR products were visualized using electrophoresis on 1.5% agarose gels, followed by purification using spin columns (Qiagen, Germany). The sample was prepared using the ABI prism terminator cycle sequencing-ready reaction kit and sequenced on an Applied Biosystems (Model 3100) automated sequencer. The identity of the 28S rRNA gene sequence was established by performing a similarity search against the sequences in the GenBank database (http://www.ncbi.nih.gov/BLAST). The sequence of ASEF14 was submitted to the National Centre for Biotechnological Information (NCBI) database, and an accession number was obtained. Using the ASEF14 sequence, a phylogenetic tree was constructed using the neighbor-joining method originally described by Saitou N and Nei M [34] in MEGA 5.0 software [35], along with the existing 28S rRNA gene sequences from the related *Aspergillus* sp. obtained from the NCBI GenBank database.

### Growth kinetics and lipid production by *A. caespitosus* ASEF14 in SM and SWW

Based on the high lipid accumulation, amylase secretion, and starch utilization in SM, the superior oleaginous fungus *A. caespitosus* ASEF14 was selected to study their physiological response and lipid accumulation kinetics in SWW as well as SM, respectively. The fungal strain was grown in SM and SWW for ten days at 30 °C. Before inoculation into SWW, the pH of the SWW was adjusted to near neutral (pH 6) with 0.1 N NaOH. Based on the processing steps in sago production, the starch content in SWW was varied (7 to 10 g L^−1^). In the present study, the raw SWW contained 6.10 g L^−1^ of starch. Hence, the initial starch concentration of SWW was adjusted to 30 g L^−1^, similar to SM.

Periodically, both fungal biomass and spent culture medium were withdrawn from the experimental culture flask for estimating biomass, lipid, starch content, and amylase activity. Cell biomass was determined after harvesting the mycelia from the culture broth using filtration through Whatman No.1 filter paper. The harvested mycelia were washed thoroughly with sterile distilled water and dried in an oven at 60 °C for 15 h [31]. Next, the lipids were extracted from the dried biomass using a 2:1 mixture of chloroform and methanol as done for determining the lipid content [32]. The spent culture medium was separated by centrifugation at 5,000 rpm for 10 min, and the supernatant was used to analyze pH, total soluble starch, and amylase activity. The total soluble starch of the culture filtrate was measured using the phenol sulfuric acid method [22]. Amylase activity was measured according to the dinitrosalicylic acid (DNS) method as described by Bernfeld [36].

### FTIR spectral analysis of fungal lipids

The FTIR spectra of standard fatty acid (FAME Mix C4-C24, Sigma-Aldrich, St Louis, MO, USA) and extracted lipids from *A. caespitosus* ASEF14 grown in SWW for ten days were recorded using ATR-FTIR spectroscopy (JASCO FT/IR-6300, Japan), with a diamond-enabled ATR sample holder and a DLaTGS detector in the spectral range of 400 to 4,000 cm^–1^. The functional components in the samples were identified qualitatively by matching the maximum peak hit (> 99.5 peak region matching) with the IR Spectral Library using the KnowItAll software (BioRad Laboratories, Munchen, Germany). The analysis of the second derivative spectra (2800–3050 cm^–1^ and 1350–1500 cm^–1^) was done by the Means-Movement method (smoothing with a convolution width of 25) using the Jasco Spectra Manager software. All analyses were done thrice to confirm the reproducibility of the data.

### Principle component analysis of FTIR data

Principal component analysis (PCA) was independently performed on two ranges, 3050–2800 cm^–1^ and 1500–1350 cm^–1^, to evaluate the changes in the fatty acid composition during the fungal growth in the XLSTAT version 6.0 software [37]. Since each range provides diverse and specific information, splitting the analysis into different ranges allows an easier interpretation of the PCA results. Further, it was compared with an individual fatty acid standard such as palmitic, stearic, oleic, and linoleic acid (Sigma-Aldrich, St Louis, MO, USA). The correlation matrix was computed on standardized spectra (zero mean and standard deviation equal to 1) and diagonalized to get eigenvectors (loadings, v) sorted according to the magnitude of the corresponding eigenvalues [38]. In all cases, the first three eigenvectors already described were more than 95% of the total variance of the data. The principal components (scores) were obtained by projecting the original spectra on the orthogonal subspace defined by the first three eigenvalues.

### Fatty acid profiling of *A. caespitosus* ASEF14 by GC-FID

To evaluate the potential utilities of fungal oil from superior lipid-yielding oleaginous fungus *A. caespitosus* ASEF14 grown in SWW as a biodiesel feedstock, its fatty acid profile was determined by gas chromatography method (GC-FID). To emphasize the nutrient effect on the FAME profile, the biomass of fungus grown from SM was also processed. For this analysis, 100 mg of biomass obtained at periodic intervals from both SM and SWW samples were separately added to a mixture of 10 mL of methanolic–sulfuric acid (0.1% sulfuric acid in methanol), followed by vigorous mixing. In this mixture, 10 mL of chloroform was added, and the resultant mixture was heated to 80 °C for 2 h. Subsequently, the samples were removed and cooled to ambient temperature. Distilled water (1 mL) was added, and the suspension was centrifuged at 1,500 rpm for 5 min. The lower aqueous phases containing the FAME were analyzed in a gas chromatography system (Perkin Elmer Clarus 680, US) coupled with a flame ionization detector (FID) using an Elite-5 column (30 m × 0.25 mm, 0.25 µm film thickness). The injection temperature was 220 °C, and the initial column temperature was 160 °C. The final temperature of 190 °C was achieved by increasing the temperature at a rate of 3 °C per min, and the detector temperature was 270 °C. Helium was used as the carrier gas at a flow rate of 1.3 mL min^–1^. The FAME composition of the fungal oil was determined by comparing the retention time and the peak area of the samples with the FAME mix (Sigma-Aldrich, St Louis, MO, USA) [14].

1 $$FAME \, composition \% = \frac{{\frac{Standard \, conc.}{{Standard area}} \times \frac{Sample\,area}{{Sample wt \left( {mg} \right) }} \times Dilution \, factor }}{10000}$$

### Fuel properties of fungal biodiesel

The fuel and quality criteria of biodiesel are strongly influenced by the chemical structure (chain length and number of double bonds) of fatty acids present in the lipid feedstock [39]. We used the predictive model equations to evaluate biodiesel fuel properties based on their fatty acid composition. To evaluate the fuel properties of SWW-grown *A. caespitosus* ASEF14, key physicochemical fuel properties, including density (ρ), kinematic viscosity (KV), saponification value (SV), iodine value (IV), higher heating value (HHV), oxidative stability (OS), cetane number (CN), long-chain saturated factor (LCSF), cold filter plugging point (CFPP), cloud point (CP), pour point (PP), and degree of unsaturation (DU), were estimated. The equations given below were used to estimate the various properties of fungal biodiesel from the FAME profile.

Kinematic viscosity (υ, mm^2^ s^–1^) at 40 °C, density (ρ, g cm^–3^) at 20 °C, and HHV of the biodiesel were estimated using the following equation [40]

2 $$\ln \left( \upsilon \right) = \sum {{\text{N}}_{{\text{i}}} \left( { - 12.503 \, + \, \left( {2.496 \times \ln {\text{ Mw}}_{{\text{i}}} } \right) \, - \, 0.178 \, \times {\text{ D}}_{{\text{i}}} } \right)}$$

3 $$\rho = \sum {{\text{N}}_{{\text{i}}} \left( {0.8463 \, + \, \left( { \, 4.9/{\text{Mw}}_{{\text{i}}} } \right) \, + \, 0.0118 \, \times {\text{ D}}_{{\text{i}}} } \right)}$$

4 $${\text{HHV}} = \sum {{\text{N}}_{{\text{i}}} \left( {46.19 - \left( {1794/{\text{Mw}}_{{\text{i}}} } \right) - 0.21 \, \times {\text{ D}}_{{\text{i}}} } \right)}$$

Here, Mw_i_ is the molecular weight of a fatty acid, N_i_ is the percentage of the given fatty acid in the biodiesel, and D_i_ is the number of double bonds in the given fatty acid.

The saponification value (SV), iodine value (IV), and cetane number (CN) [41] were calculated using the following equations:

5 $${\text{SV}} = \sum {\left( {{56}0 \times {\text{N}}} \right)/{\text{M}}}$$

6 $${\text{IV}} = \sum {\left( {{254} \times {\text{D N}}} \right)/{\text{M}}}$$

7 $${\text{CN}} = 46.3 + \left( {5.458/{\text{SV}}} \right) - \left( {0.225 \times {\text{IV}}} \right)$$

where D = number of double bonds in the fatty ester, M = molecular mass of fatty ester, and N = percentage of the particular fatty ester in oil sample. The degree of unsaturation (DU) and oxidative stability (OS) [42] were calculated as follows:

8 $${\text{DU}} = {\text{MUFA}} + \left( {{2} \times {\text{PUFA}}} \right)$$

9 $${\text{OS}} = {117}.{9295}/\left( {{\text{wt}}\% {\text{ C}}_{{{18}:{2}}} + {\text{wt}}\% {\text{ C}}_{{{18}:{3}}} + {2}.{59}0{5}} \right)$$

where MUFA = monounsaturated fatty acid and PUFA = polyunsaturated fatty acid.

The LCSF, CFPP, CP [43], and PP [44] were estimated as follows:

10 $${\text{LCSF}} = \left( {0.{1} \times {\text{C}}_{{{16}}} } \right) + \left( {0.{5} \times {\text{C}}_{{{18}}} } \right) + \left( {{1} \times {\text{C}}_{{{2}0}} } \right) + \left( {{1}.{5} \times {\text{C}}_{{{22}}} } \right) + \left( {{2} \times {\text{C}}_{{{24}}} } \right)$$

11 $${\text{CFPP}} = \left( {{3}.{1417} \times {\text{LCSF}}} \right) - {16}.{477}$$

12 $${\text{CP}} = \left( {0.526 \times {\text{C}}_{16} } \right) - 4.992$$

13 $${\text{PP}} = \left( {0.{571} \times {\text{C}}_{{{16}}} } \right) - {12}.{24}$$

### Decontamination study

After kinetic fermentation, the treated/spent wastewater was filtered and used to estimate the physicochemical parameters, similar to raw wastewater. The percentage reduction was calculated and compared.

### Statistical analysis

All experiments were performed in triplicate. Data are presented as mean ± standard deviation (*n* = 3). The statistical significance between the samples was evaluated using one-way ANOVA (analysis of variance) (*p* < *0.05*) in the XLSTAT version 6.0 software.

## Results and discussion

Significant amounts of starch are present in tapioca tubers. Its industrial processing generates enormous wastewater that is rich in organic loads and starch. Therefore, it is highly important to explore such wastewater as a substrate for lipid production by oleaginous fungi. Hence, we isolated starch-utilizing fungi having a high lipid-accumulating ability, which could be used for biodiesel production and simultaneous decontamination of starch-rich wastewater.

### Isolation and screening of fungal isolates for amylase activity

**Fig. 2:**
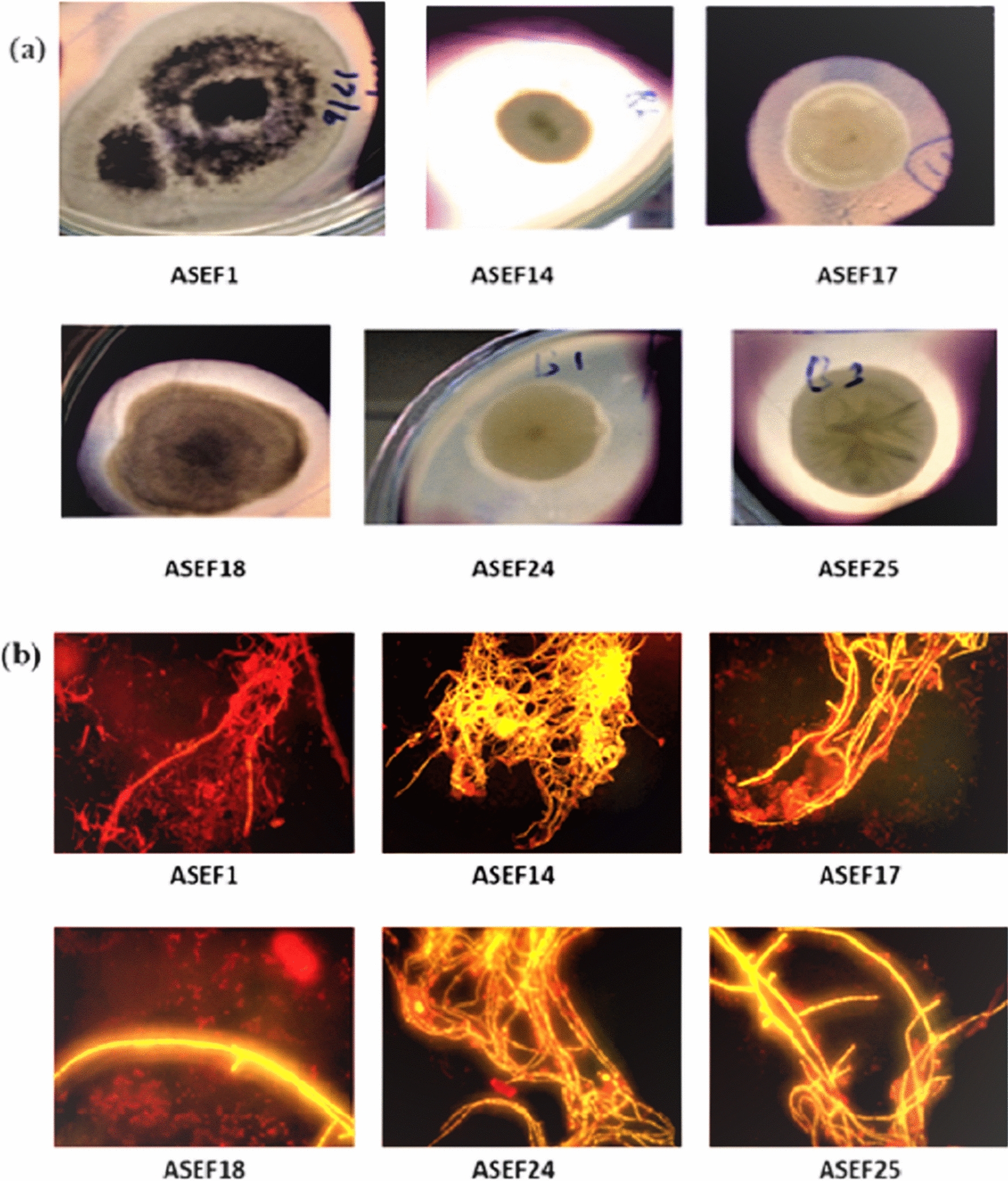
**a** Amylase-positive isolates showing a clear zone around the colony on starch agar. **b** Nile red-stained lipid bodies of selected fungal isolates

Fifteen fungal isolates (ASEF1, ASEF2, ASEF4, ASEF7, ASEF8, ASEF9, ASEF10, ASEF12, ASEF13, ASEF14, ASEF15, ASEF17, ASEF19, ASEF20, and ASEF23) with the different visible colony and cell morphology (as observed under the microscope) were obtained from the enriched SWW sample. Amylase is the primary enzyme responsible for starch hydrolysis. It is reported that when complex carbon sources are used for microbial lipid production, the capability of oleaginous fungi to secrete amylase is critical to obtain high lipid yields [45, 46]. As SWW is rich in starch, screening the fungal isolates for amylase activity would be a prerequisite for assessing their ability to efficiently grow and produce lipids [47]. Hence, the selected isolates were screened for amylase production using the starch plate method, resulting in a clear zone of starch hydrolysis in the Petri dishes after iodine treatment. Among them, six fungal isolates were found to be positive for amylase production, as determined by measuring the width of the clear zone (zone of hydrolysis) formed around the fungal colonies on starch agar medium, and its width ranged from 8 to 35 mm (Fig. 2a). The fungal isolate ASEF14 produced the maximum zone of hydrolysis, i.e., 35 mm, followed by ASEF24 (30 mm) and fungal isolate ASEF1 produced a minimum zone of 8 mm (Fig. 2a). These isolates were selected for further screening of oleaginicity by NR staining. Quantitative amylase assay revealed the maximum amylase activity of 48.03 IU mL^–1^ for the fungal isolate ASEF14.

### Nile red staining

High levels of lipid accumulation were observed in oleaginous fungi when carbon was available in excess, whereas critical nutrients such as nitrogen or phosphorus were limited [48]. These accumulated lipids or single-cell oils (SCO) are deposited as intracellular LBs. NR, a fluorescent lipophilic probe, was used to stain and detect LBs in intact cells [49]. This dye has great advantages over other dyes due to its fluorescence emission spectrum, which differs according to the type of lipid. Lipid detection by NR has been commonly measured with excitation at 480 to 490 nm and 510 to 560 nm. The former target neutral lipids showed yellow emission, and the latter target polar lipids showed red or orange emission [50]. Such differentiation enabled the efficient use of NR to select microorganisms that accumulated lipids of interest for biodiesel production. The yellow emission was observed throughout the fungal hyphae of ASEF14, ASEF17, ASEF18, ASEF24, and ASEF25 isolates, which confirmed their oleaginicity and the presence of neutral lipids (Fig. 2b). In contrast, a faint or no fluorescence signal was detected in the case of non-oleaginous fungal isolate ASEF1 (Fig. 2b), while in control, red color was seen. Kimura et al. [28] visualized the LBs of several oleaginous fungi, namely, *Lipomyces starkeyi* IFO–10381, *Rhodosporidium toruloides* IFO–0559, *Cryptococcus curvatus* IFO–1159, and *Mortierella isabellina* IFO-7884 that varied in size, number, and shape [28].

### Biomass and lipid yields (SCOs)

The application of oleaginous microorganisms for oil production necessitates the use of strains with high lipid-accumulation capability. All six fungi grew well in a carbon-rich, nitrogen-limiting medium for up to seven days [45]. They significantly differed in their biomass yield, lipid yield, and lipid content. All fungal cultures produced good biomass with the dry cell weight (DCW) ranging from 4.10 to 9.01 g L^–1^. The total lipid from all fungal cell mass was extracted and estimated (Table 1). The fungal isolates varied in their lipid yield; the ASEF14 culture produced the maximum lipid yield of 3.31 g L^–1^. Among the six fungi, three were found to be oleaginous as the total lipid contents were more than 20%, with the highest lipid content of 54.6% for ASEF14, followed by 28.51% for ASEF25 in SM. The results of the present study are similar to those of previous reports that stated, the lipid-producing potential of fungi such as *Alternaria alternata* (40.7% lipid content per dry mass), *Cladosporium cladosporioides* (38.5%), *Epicoccum nigrum* (38%), *Fusarium oxysporum* (32%), *Aspergillus parasiticus* (28.2%), *Emericella nidulans* var*. lata* (24.5%), *Mucor circinelloides* MU241 (23%), *Aspergillus* sp. (23.3%), and *Schizochytrium* sp. LU310 (49%–67%) [29, 47, 51, 52].

**Table 1:** Microbial lipid production by selected fungal isolates

Isolates	Biomass yield (g L^–1^)	Lipid yield (g L^–1^)	Lipid content (%)
ASEF1	6.66 (± 1.0)^b^	1.04 (± 0.1)^c^	16.29 (± 4.0)^bc^
ASEF14	6.06 (± 0.0)^bc^	3.31 (± 0.3)^a^	54.6 (± 5.0)^a^
ASEF18	9.01 (± 1.0)^a^	1.60 (± 0.0)^b^	17.97 (± 2.0)^bc^
ASEF17	4.10 (± 0.4)^c^	0.95 (± 0.0)^c^	23.29 (± 1.3)^bc^
ASEF24	4.73 (± 0.0)^bc^	0.67 (± 0.1)^c^	14.16 (± 2.1)^c^
ASEF25	5.33 (± 0.2)^bc^	1.52 (± 0.0)^b^	28.58 (± 1.8)^b^

### Identification of superior lipid-yielding fungal isolate (ASEF14)

**Fig. 3:**
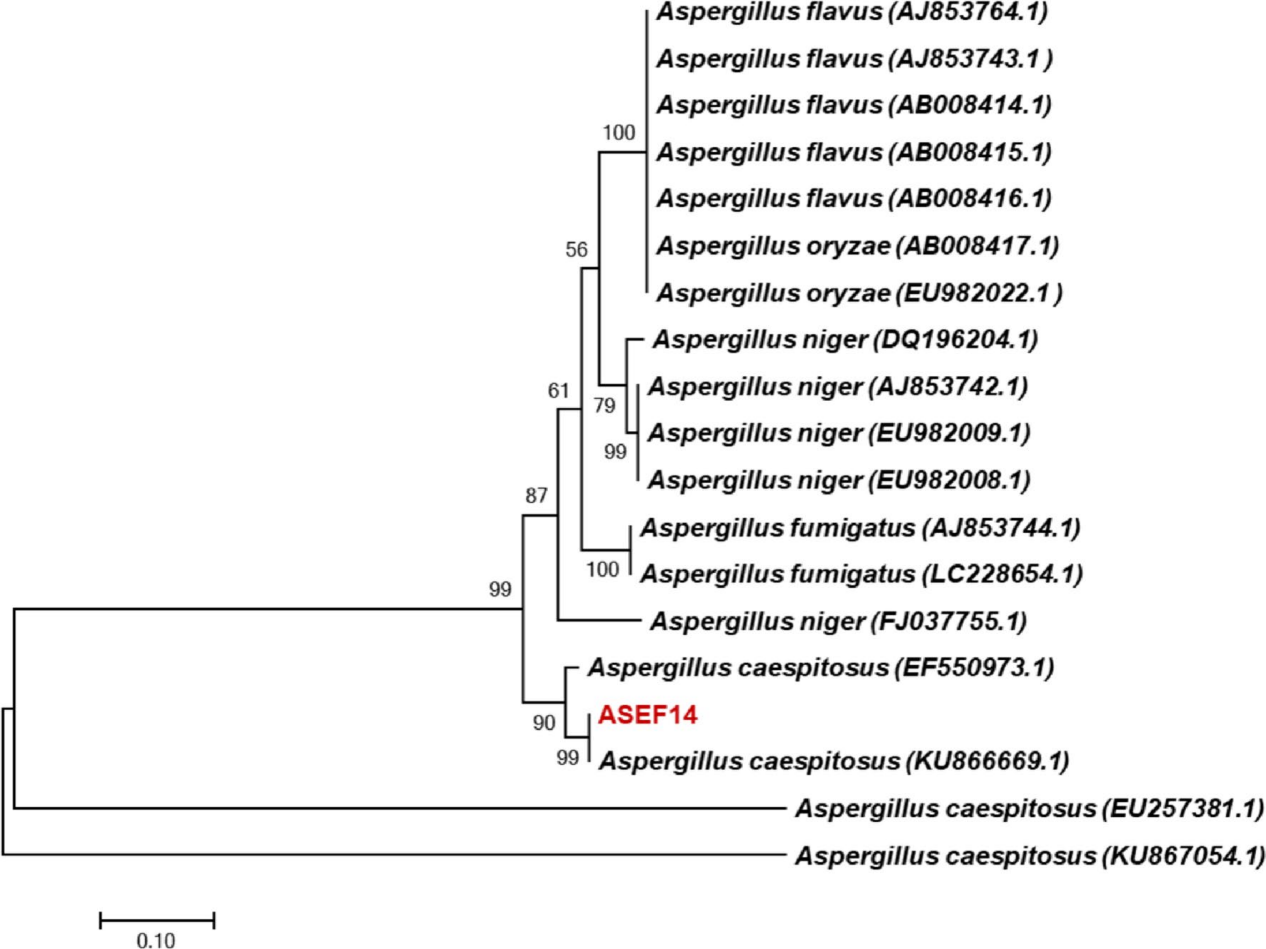
Phylogenetic tree based on the 28S rRNA gene sequence from hyper lipid-yielding oleaginous isolates *A. caespitosus* ASEF14 from SWW using the neighbor-joining method. The data of other 28S rRNA gene sequences of *Aspergillus* spp. were obtained from GenBank (accession numbers are given in parentheses). The numbers above or below branches indicate bootstrap values (> 50%) from 1000 replicates. The horizontal bar at the bottom represents evolutionary distance as 0.01 changes per nucleotide position, determined by measuring the lengths of the horizontal lines connecting the species

The total DNA of the maximum lipid-producing fungus ASEF14 was isolated. The ITS region of the isolate was PCR amplified using ITS1 and ITS4 primers [53]. The PCR product showed an amplicon band size of 600 bp. The analysis of the ASEF14 sequence using BLAST showed 100% similarity with *A. caespitosus.* The 28S rRNA gene sequence data of the above strain is available in the NCBI database with accession no. MF599090. Phylogenetic analysis using the neighbor-joining method positioned and clustered the fungus strain with corresponding sequence-matching genomes (Fig. 3).

Understanding the mechanisms driving lipogenesis could have a major impact on the future use of SCOs as biodiesels. The information available on lipid metabolism in *Aspergillus* spp. at the cellular level is very limited. In 2012, Vorapreeda et al. [54] compared the genomes of non-oleaginous (*Saccharomyces cerevisiae*, *Candida albicans*, and *Ashbya gossypii*) and oleaginous strains (*Yarrowia lipolytica*, *Rhizopus oryzae*, *Aspergillus oryzae*, and *Mucor circinelloides*) and discovered 209 orthologous protein sequences; of these, 41 sequences were involved in the production of acetyl-CoA, a precursor for fatty acid and lipid synthesis, but they were absent in non-oleaginous strains. It was also suggested that there exists a link between carbohydrate, lipid, and amino acid metabolism.

Thammarongtham et al. [55] reported the genome sequence of the oleaginous *A. oryzae* BCC7051. They compared the structural genes focusing on the involvement in lipid metabolism among oleaginous yeast and fungi, which revealed the presence of multiple isoforms of metabolic enzymes responsible for fatty acid synthesis. They also found alternative routes of acetyl-CoA generation and malate/citrate/pyruvate shuttle as oleaginous features. This shuttle mechanism appears to be universal in eukaryotes [56, 57], but the exact metabolic network of this shuttle is species-specific. The analysis of the genomic data aids in comprehending the species’ distinctive characteristics for high-level lipid accumulation, suggesting the *Aspergillus* genome could be a potential candidate for biodiesel production.

### Growth and lipid production kinetics of *A. caespitosus* ASEF14 grown in SM and SWW

**Fig. 4:**
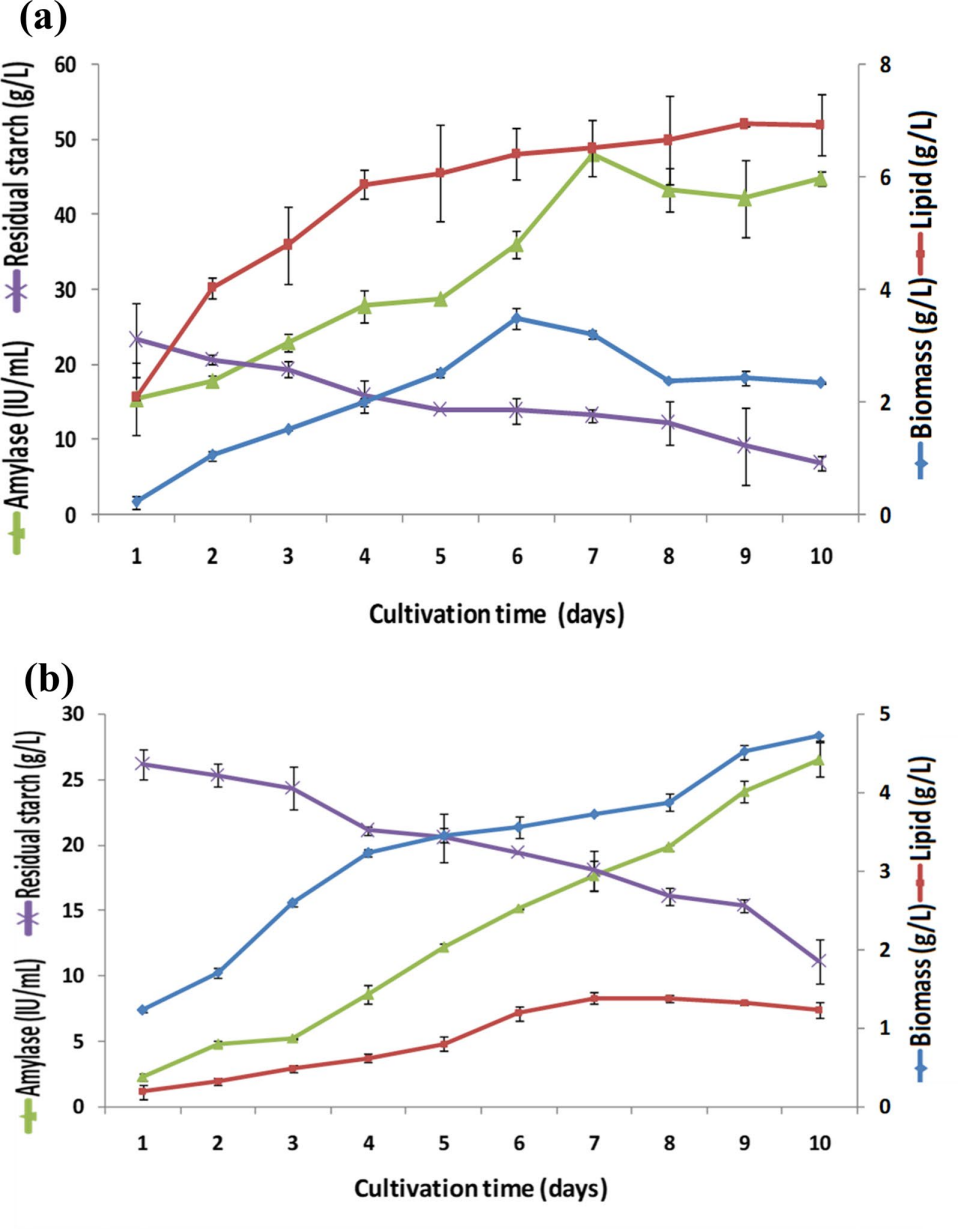
**a** Kinetics of growth and lipid production of *A. caespitosus* ASEF14 in SM. **b** Kinetics of growth and lipid production of *A. caespitosus* ASEF14 in SWW

*A. caespitosus* ASEF14 was grown in SM and SWW with a C:N ratio of 30:1 for ten days. The samples were withdrawn at every 24 h interval for up to ten days. In SM, the fungus produced the maximum biomass of 6.93 g L^–1^ on the 10^th^ day, with a lipid yield of 2.35 g L^–1^ and the highest starch utilization of about 23.07 g L^–1^. However, the maximum lipid yield of 3.50 g L^–1^ was observed on the sixth day, with starch utilization of about 16.01 g L^–1^ (Fig. 4a). The maximum amylase activity of 48 IU mL^–1^ was observed on the seventh day; however, after the eighth, ninth, and tenth days of incubation, only little variability was observed in the values (Fig. 4a).

In SWW, the fungus attained the maximum biomass of 4.73 g L^–1^ on the 10^th^ day, with a lipid yield of 1.23 g L^–1^. Nevertheless the fungus produced the maximum lipid yield of 1.39 g L^–1^ and lipid content of 37.27% on the seventh day. Afterward, the lipid yield and content declined gradually. The lipid content of more than 30% was recorded on sixth (33.5%), seventh (37.27%), and eighth days (35.37%). The amylase secretion gradually increased from the first day onwards and attained the maximum value on the 10^th^ day (26.51 IU mL^–1^). The residual starch level at the end of fermentation was 11.12 g L^–1^. The kinetic analyses of *A. caespitosus* ASEF14 grown in SWW are presented in Fig. 4b.

The lipid yield reported in this study seems to be promising in the cost-effective production of lipids from SWW, and there is plenty of scope for improvement by optimizing the nutritional and growth conditions. The lipid content of ASEF14 was found to be around 37.27%, which is consistent with the following findings. The oleaginous yeast *R. glutinis* cultured on corn starch wastewater supplemented with waste syrup has a lipid content of 35%, according to Xue et al. [58]. Muniraj et al. [13] reported that oleaginous fungus grown on PPW produced a lipid content of 40%.

The conversion rate or Y_L/C_ (g of lipid produced per g of substrate consumed) in SM by *A. caespitosus* ASEF14 was found to be 0.03 to 0.22 g of lipid per gram of starch consumed. The maximum Y_L/C_ value of 0.22 g g^–1^ was obtained on day 6 of fermentation. In SWW, Y_L/C_ values ranged from 0.05 to 0.12 g g^–1^ of starch consumed. When microalgae *Schizochytrium limacinum* was grown on biodiesel-derived glycerol, a similar lipid yield of 0.15 g lipids per gram of glycerol was produced [59]. *Cunninghamella echinulata* ATHUM 4411, an oleaginous fungus, converted a gram of starch into 0.15 g lipids when cultivated on pure starch [45]. Muniraj et al. [13] found that *A. oryzae* cultured in PPW yielded 0.11–0.16 g lipids per gram of soluble starch. In our prior investigation, the oleaginous yeast *Candida tropicalis* ASY2 produced 0.079–0.135 g of lipid per gram of starch consumed [14]. This is noteworthy because the lipid yield on glucose consumption can seldom exceed 0.22 g lipids per gram of glucose under optimal circumstances for SCO synthesis [60]. Hence, the lipid accumulation potential of the studied oleaginous fungi seems to be much better.

The amylolytic potential of fungus was directly associated with the Y_L/C_ values. The high amylase enzyme secretion by fungi in the SM (48 IU mL^–1^) boosted substrate utilization by two-fold (Y_L/C_: 0.22 g g^–1^) compared to SWW (26.51 IU mL^–1^; 0.12 g g^–1^). The higher substrate conversion seen in SM could be attributed to the existence of a more easily degradable substrate (soluble starch). In SWW, the substrate was available either in aggregated/complex (waste solids) or dissolved form.

Fungi produce enzymes that are specific to the substrates that aid in the breakdown of complex molecules into simple forms, and it is used as a metabolic form. A filamentous fungus *Aspergillus* capable of producing glucoamylases, pectinases, and galactosidases from complex molecules [46]. In the present study, we also confirmed that the amylolytic potential of oleaginous fungi is linked to the consumption of its relevant substrate starch. This promotes chemo-heterotrophic fungal nutrition, resulting in increased growth and lipid accumulation.

### FTIR analysis of lipid profile change in oleaginous fungi during lipid accumulation

**Fig. 5:**
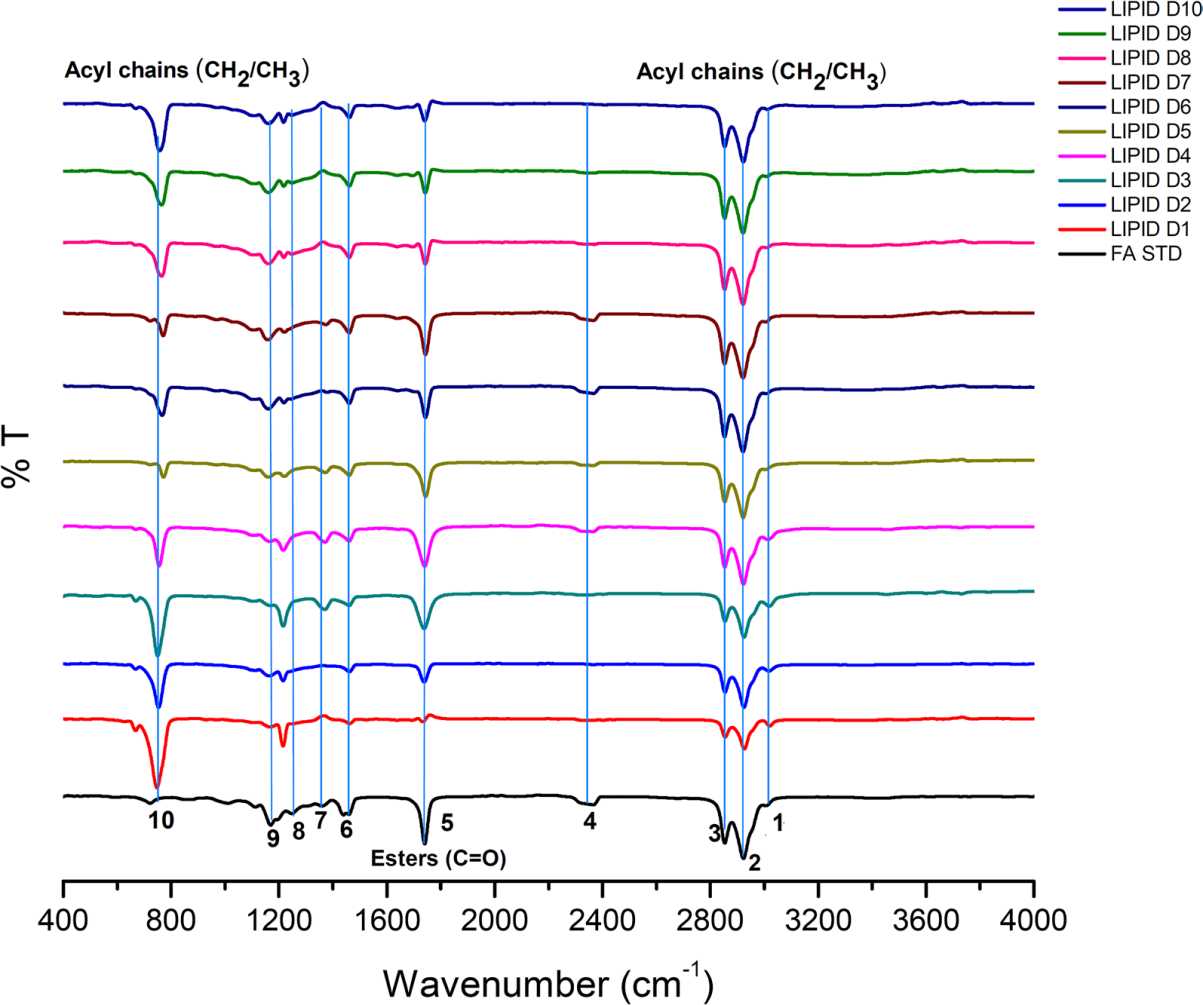
Fourier transform infrared (FTIR) analysis of *A. caespitosus* ASEF14 lipid samples derived from day 1 to 10 grown in SWW. The most important lipid molecule absorption peaks are indicated (1–10)

The spectra collected from cells are in their natural state and are considered to be a molecular “fingerprint” of the total chemical composition of cells. As a result, they form a distinct fingerprint of cell lipids, proteins, nucleic acids, and carbohydrates [61, 62]. In the present study, the changes in the lipid profile of *A. caespitosus* ASEF14 from day 1 to day 10 grown in SWW were investigated using FTIR spectroscopy (Fig. 5). Table 2 lists the assigned FTIR spectral bands and their corresponding functional groups.

**Table 2:** Peaks assignment in the FTIR spectra of microbial lipid (lipid characteristics)

Peak No	Wavenumber (cm^–1^)	Peak assignment	Representing biomolecule	References
1	3008	=C–H stretching	Lipid	[85, 86]
2	2953	–C–H (CH_3_) stretching (asym)	Lipid	
3	2924	–C–H (CH_2_) stretching (asym)	Lipid	
4	2853	–C–H (CH_2_) stretching (sym)	Lipid	
5	1745	–C=O (ester) stretching	Lipid	
6	1465	–C–H (CH_2_, CH_3_) bending (scissoring)	Lipid	
7	1415	C–H rocking	Protein	[87]
8	1377	–C–H (CH_3_) bending (sym)	Lipid	[86]
9	1240–1265	P=O asymmetric stretching of > PO_2_ phosphodiester	Polyphosphate, phospholipid	
10	720	CH_2_ rocking, bending	Lipid	

In these infrared spectra, cellular lipids were represented by several peaks, related to different lipid functional groups: (i) peaks in the regions 3050–2800 cm^−1^ (peaks: 1–3 in Fig. 5), (ii) 1500–1300 cm^−1^ (peaks: 6–9), and (iii) at 725 cm^−1^ (peak: 10) are related to lipid acyl chains. In detail, the absence of peaks from 4000 to 3010 cm^−1^ region in the extracted lipid indicated the absence of a free hydroxyl group (–OH) and an amine group (–NH_2_). The peak around 3008 cm^−1^ (peak: 1) corresponds to = C–H stretching and indicates lipid unsaturation index of lipids and oils [63]. In the fatty acids of TAGs, peak at 2955 cm^−1^ and 1380 cm^−1^ representing CH_3_ stretching of acyl chains; peaks at 2925 cm^−1^, 2850 cm^−1^, and 725 cm^−1^ representing CH_2_ stretching of acyl chains (Fig. 5). The other important peak for the lipid analysis occurs in the lower wavenumber region at 1745 cm^−1^ (peak: 5), which is related to the ester carbonyl bond from lipid triglycerides and fatty acids, which represents the majority of the total lipid content in the cell [4, 63].

In detail, when comparing the spectra of *A. caespitosus* ASEF14 from day 1 to day 10, we observed significant differences, owing to the accumulation of lipids. Indeed, the intensity of the CH stretching bands spanning around 3050 and 2800 cm^–1^ increased until the middle of the fermentation days (days 1–7), then began to decline slightly. It may be due to stress response or lipid turnover. On day 2, the existence of unsaturated fatty acids is due to the olefinic group = CH at 3010 cm^–1^ in the IR spectrum.

From day 2 onward, the strength of the ester carbonyl band about 1740 cm^–1^ increased dramatically. Overall, these findings confirmed the intensification of all lipid-related bands in mid-IR spectra of *A. caespitosus* ASEF14 with the cultivation time leading to major biochemical changes in the fungus that were related to intracellular lipid accumulation.

### Second derivative analysis of the FTIR fungal spectra in lipid region

**Fig. 6:**
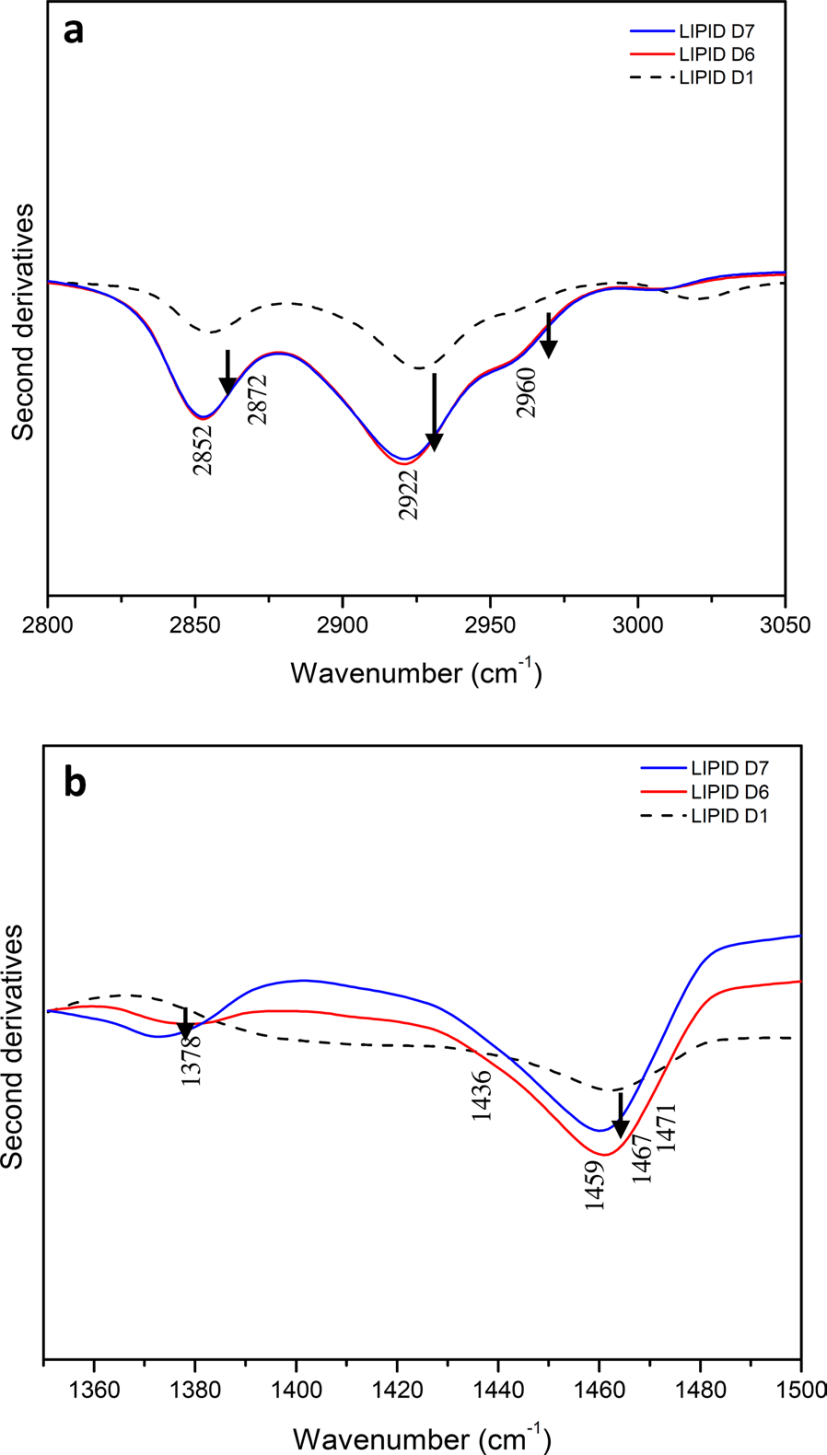
Second derivative analyses of *A. caespitosus* ASEF14 lipid samples. **a** The second derivative spectra are reported between 3050 and 2800 cm^–1^ wavelength range and **b** 1500–1350 cm^–1^, after normalization. The arrows represent increasing intensities

We studied the second derivatives of the FTIR absorption spectra to understand better the spectral changes in the lipid component that occurs throughout the growth phase because it enabled resolving the overlapping components of IR absorption bands [4]. In particular, we compared the second derivative spectra of extracted lipid from day 1 to day 6 and 7, when the oleaginous fungi had stored a significant amount of FA. Figure 6a shows the second derivative spectra in the range of 3050 to 2800 cm^–1^, which is dominated by lipid acyl chain absorption. As shown in Fig. 6a, the spectrum on day 1 is characterized mainly by four bands at approximately 2960 cm^–1^ (ν_asym_ CH_3_), 2922 cm^–1^ (ν_antisym_ CH_2_), 2872 cm^–1^ (ν_sym_ CH_3_), and 2852 cm^−1^ (ν_sym_ CH_2_). These spectral features changed dramatically on days 6 and 7; in particular, the intensity of the acyl chain bands increased. Furthermore, due to the = CH stretching mode, which is typical of unsaturated fatty acids, a well-resolved component was nearly non-existent on day 1 and appeared at 3009 cm^–1^ on days 6 and 7. Overall, these findings suggested that the lipid content changed significantly during their growth, likely reflecting the accumulation of fatty acids at high levels, including unsaturated ones.

The absorption between 1500 and 1350 cm^–1^ (see Fig. 6b) was subsequently investigated, because this is where other vibrational modes related to lipid CH_2_ and CH_3_ occur. In particular, the second derivative spectrum of *A. caespitosus* ASEF14 on day 1 was characterized by well-resolved components around 1471 cm^–1^ (acyl chain CH_2_ bending and/or CH_3_ deformation), 1459 cm^–1^ and 1436 cm^–1^ (CH_3_ deformation) [64, 65]. On days 6 and 7, new well-resolved components attributable to CH_3_ bending vibrations appeared at 1378 cm^–1^, in addition to a significant rise in the intensity of the 1467 cm^–1^ band [66]. These spectral fluctuations indicate changes in the metabolism associated with lipid synthesis during growth. FTIR spectroscopy can reveal small variations in cultivation parameters such as variations in culture time, medium composition, pH, temperature, water content or culture storage conditions [62, 67].

Ami et al. [4] used FTIR for the comparison of lipid accumulation of two oleaginous yeasts and the non-oleaginous species *Saccharomyces cerevisiae*. Deeba et al. [68] monitored the lipid profile of oleaginous yeasts by FTIR for extracted lipid samples. Signori et al. [69] monitored the lipid accumulation from crude glycerol over the fermentation time in different oleaginous yeasts. Shapaval et al. [70] characterized the total biochemical profile of oleaginous yeasts, which enabled us to identify strains and substrate(s) providing the highest total lipid content using FTIR spectroscopy. In the present study, FTIR spectroscopy successfully demonstrated the lipid accumulation pattern over the fermentation time in oleaginous fungi *A. caespitosus* ASEF14 from SWW.

### FTIR spectroscopy coupled with principal component analysis (PCA) for monitoring lipid accumulation

**Fig. 7:**
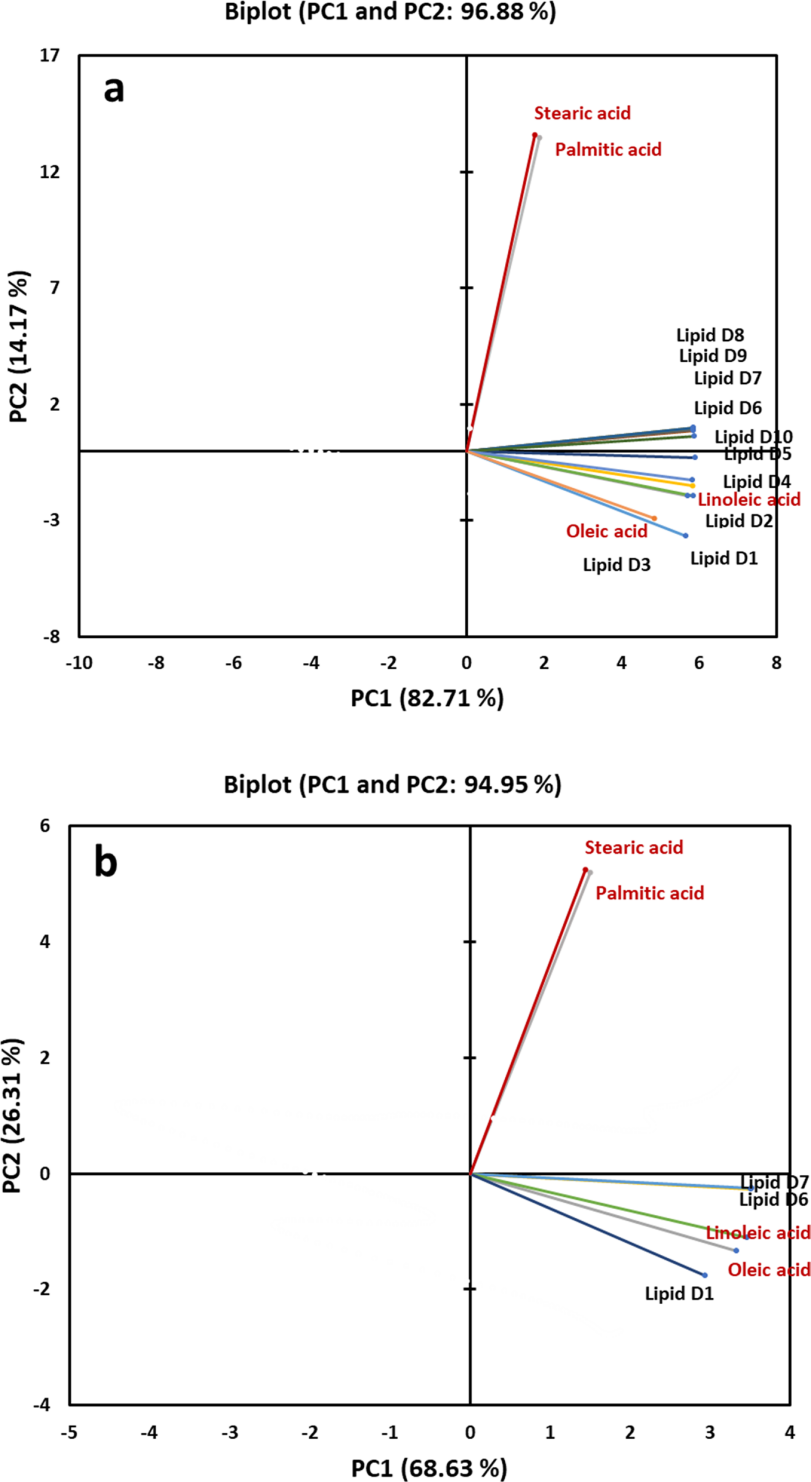
PCA two-dimensional score plots of *A. caespitosus* ASEF14 lipid samples and of the selected fatty acid standards, performed between 3050 and 2800 cm^–1^. **a** Lipid samples from day 1 to day 10 **b**. Lipid samples from day 1 and day 6 & 7. PC1 and PC2 are reported. PCA was performed on the pre-processed FTIR data (PC: principal component)

**Fig. 8:**
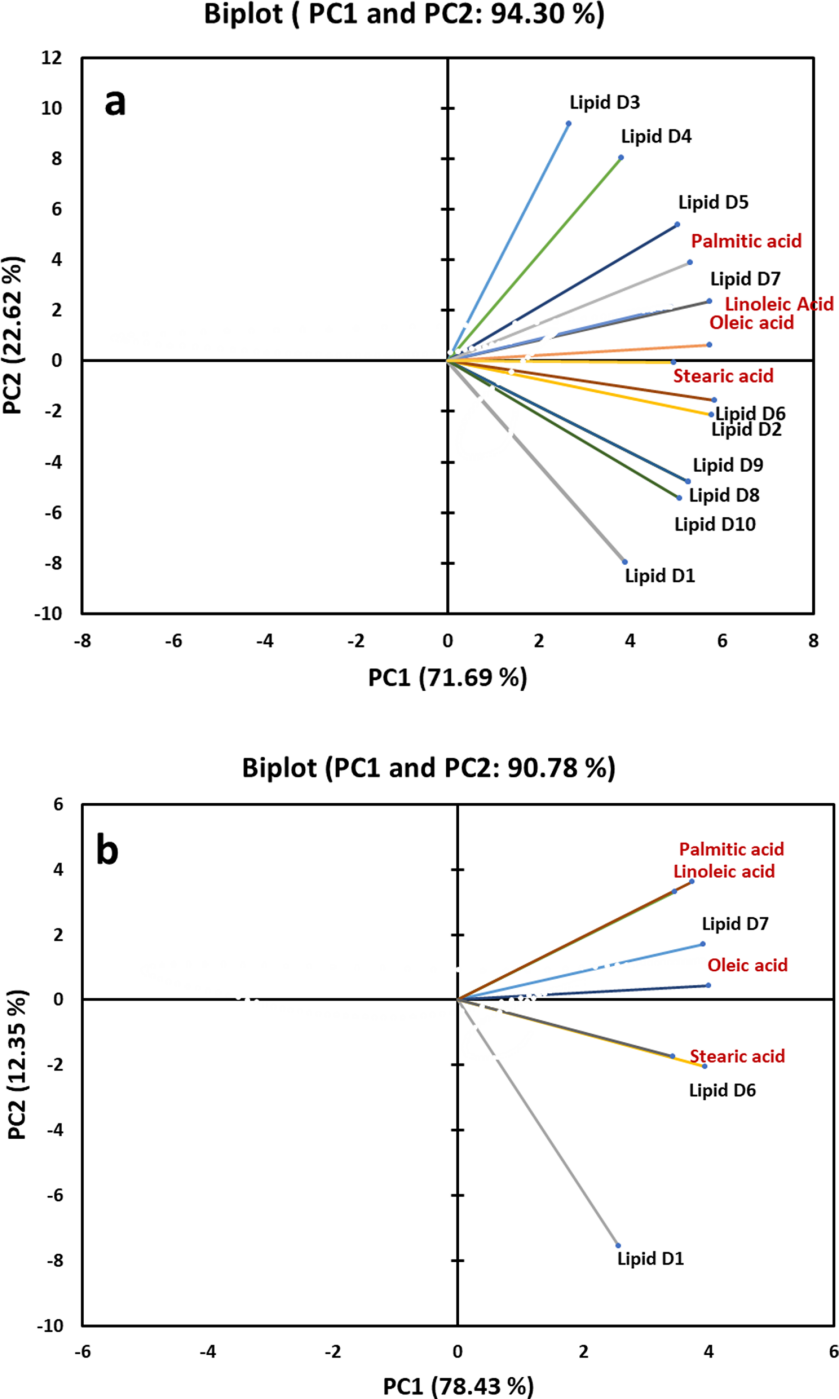
PCA two-dimensional score plots of *A. caespitosus* ASEF14 lipid samples and of the selected fatty acid standards, performed between 1500 and 1350 cm^–1^. **a** Lipid samples from day 1 to day 10 **b**. Lipid samples from day 1 and day 6 & 7. PC1 and PC2 are reported. PCA was performed on the pre-processed FTIR data (PC: principal component)

In order to determine the spectroscopic changes observed in specific lipid molecules during the growth of *A. caespitosus* ASEF14, their IR responses were compared with selected fatty acid standards, which included saturated (palmitic and stearic acid), monounsaturated (oleic), and polyunsaturated (linoleic) ones. We performed PCA for the lipid samples taken from day 1 to day 10 of *A. caespitosus* ASEF14 in two wavelength ranges, namely 3050 and 2800 cm^–1^ (Fig. 7) and 1500 and 1350 cm^–1^ (Fig. 8). The initial spectral range is predominated by the lipid acyl chain absorption, and the latter one is dominated by several vibrational modes due to both lipid acyl chains and head groups.

The PCA of the assessed variables in the range of 2800–3050 cm^–1^ showed that the first and second components explain 96.88% of the total variance among different incubation days. Of which, PC1 contributes 82.71%, while PC2 adds another 14–17% (Fig. 7a). The bi-plots describing the orthogonal positions of variables explained by the first two PCs are presented in Fig. 7a. In particular, looking at the PCA score plots, we observed that lipid samples derived from days 1 and 5 were plotted in the F3 along with individual standard fatty acids, namely oleic and linoleic, but days 6 to 10 samples were plotted in F4. This may be due to shorter vector length than the other day variables. In the samples of all studied days, oleic and linoleic standard fatty acids were positively correlated with each other (narrow angled) due to unsaturation represented by CH_2_ (ν_sym_) and =CH, likely reflecting a higher amount of unsaturated fatty acids accumulation. The standards stearic and palmitic fatty acid observed at its right angle did not correlate with other days’ lipid sample.

Further, to observe a clear time-dependent lipid profile behavior in *Aspergillus*, the samples were taken only at days 1, 6 and 7. Interestingly, as shown in Fig. 7b, the lipid samples of fungus at day 1 were well separated from the samples at day 6 and 7 (right angled), indicating that during the growth, the lipid spectral features changed more significantly. The relationship with standard fatty acid was the same, as mentioned earlier.

We then analyzed the absorption between 1500 and 1350 cm^–1^ (Fig. 8), where other vibrational modes due to lipid CH_2_ and CH_3_ occurred. The PCA of the assessed variables showed that the first and second components explain 94.30% of the total variance among the different incubation days. Of which, PC1 contributes 71.69%, while PC2 adds another 22.62% (Fig. 8a). This PCA score plots were distinct from the previous wave ranges. Day 1 sample was significantly different from the other day samples. The lipid samples at days 6 and 2 were closely related with fatty acid standard stearic acid. Day 7 sample overlapped with linoleic acid and was slightly related to palmitic and oleic fatty acids. The high-level lipid accumulation of these fatty acids was observed on these days. Day 8, 9, and 10 samples were clustered together, indicating less variation in the lipid profile [4, 69].

Further PCA was carried out only for the lipid samples of days 1, 6, and 7. Interestingly, the analysis between 1500 and 1350 cm^–1^ highlighted close connectivity of stearic acid with day 6 lipid sample (Fig. 8b). Day 7 samples were narrow angled with all other fatty acids except for stearic acid. In all cases, the results confirmed that the content of all analyzed fatty acids increased during cell growth and that the increase was again higher in mid incubation days.

### Fatty acid profiling of *A. caespitosus* ASEF14 grown on SM and SWW by GC-FID

The utilization of low-cost substrates, in addition to direct *in-situ* one-step conversion of lipid-containing microbial cell mass to FAMEs, is another possible option to improve the process economics of microbial biodiesel synthesis. In the present study, renewable starch-rich SWW streams were used to produce a cost-effective fuel feedstock and platform chemicals by fermentation. In oleaginous fungi, the chain length and degree of unsaturation of fatty acids depend on the growth substratum used. The diverse metabolic processes in the microorganism help to uptake the different growth substrates (as carbon sources) from the environment that can lead to diverse fatty acid composition [71]. In the present study, the differences in both the composition and concentration of fatty acids were observed in the FAME profile when the fungal strain was grown on a simple medium (SM) and a complex substrate (SWW).

**Fig. 9:**
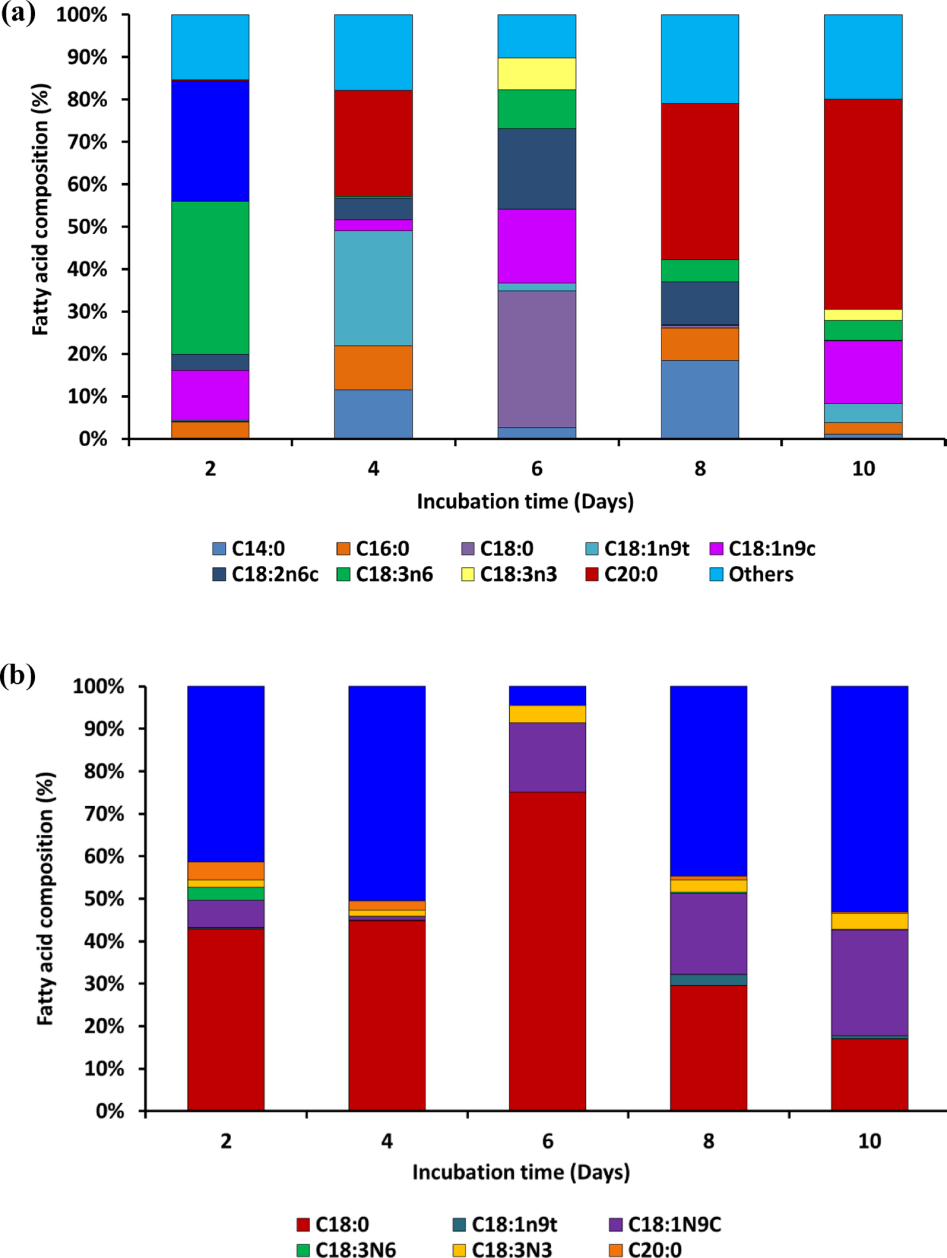
**a** Fatty acid composition of *A. caespitosus* ASEF14 grown in SM. **b** Fatty acid composition of *A. caespitosus* ASEF14 grown in SWW

When grown on SM, the fungus produced various ratios of saturated (SFA), monounsaturated (MUFA), and polyunsaturated fatty acids (PUFA) at different incubation days. Methyl palmitate (C16:0), methyl stearate (C18:0), and methyl arachidate (C20:0) were the main saturated fatty acids. The monounsaturated components were dominated by methyl elaidate (C16:1) and palmitoleate (C17:1) (Fig. 9a). The most common PUFAs were methyl linoleate and methyl linolenate. The fungi produced a significant amount of essential fatty acids such as gamma and alpha linolenate methyl esters (36.18% and 28.20%, respectively) on day 2 of incubation, followed by methyl oleate (11.67%). The linolenic acid level of microbial oil should not exceed 12%, if it is used as a biodiesel feedstock [13]. As a result, the lipid produced on this day is not acceptable as a biodiesel feedstock, but it can be used for nutritional purposes. The C16 and C18 methyl ester series were observed on days 4 to 8 of incubation. On day 6 (17.29%) and 10 (14.87%), the methyl oleate level was high, whereas the methyl linoleate (C18:2) concentration was high only on day 6 (19.12%). On days 4, 6, and 8, myristic and palmitic acids were present at significant levels; however, stearic acid (C18:0) was detected only on day 6 at 32% level (Fig. 9a). With the exception on day 4, the elaidic acid content (C18:3) was insignificant on all days, whereas PUFAs with four or more double bonds were not observed. These results suggested that a varying composition of fatty acids was produced at different incubation times by the fungal strain ASEF14, depending on the growth conditions and nutrient availability.

The fungal FAMEs obtained in SWW were rich in SFA than PUFA. The lipid profile exhibited that methyl stearate was the major fatty acid, with the highest amount of 64.77% produced on day 6 followed by day 4 (43.92%) and 2 (42.23%). The other major fatty acid methyl ester identified was methyl oleate, which was higher on day 10 (24.85%) followed by days 8 and 6 (Fig. 9b). The results of the FAME profile were also confirmed by FTIR and PCA analysis, where day 6 sample completely overlapped with stearic acid and showed a close correlation with oleic acid in the spectral range of 1500–1350 cm^–1^.

In SWW, the proportions of oleic (C18:1) and stearic (C18:0) fatty acids were two to three times higher than other fatty acids. Throughout the incubation period, there was a low amount of methyl palmitate. This fatty acid is a key precursor in the chain elongation stage for the synthesis of other fatty acids, and it was present in very meager quantities on all incubation days in the present study. Subash and Mohan [72] obtained similar results when they cultivated *Aspergillus* sp. on corncob waste liquid and produced a FAME profile rich in stearic acid (48–57%) and lacking in palmitic acids.

Muniraj et al. [17] cultured *A. flavus* and *M. rouxii* in PPW for 216 h, yielding a FAME profile of myristic acid (4% and 6%), palmitic acid (12.4 and 10.2%), palmitoleic acid (14.3 and 13.0%), stearic acid (20.2 and 17.2%), oleic acid (38.4 and 40.2%), and linolenic acid (6.8 and 8.4%). The oleic acid was the most predominant one.

The FAME profile of fungi from the present study was compared with the FAME profile of *Aspergillus* sp. cultured on several agro-industrial wastes in different studies and two commonly used vegetable oils, soybean and rapeseed (Table 3). The lipid yield and fatty acid profile of oleaginous microorganisms vary in response to environmental conditions such as carbon supply, pH, and temperature, as well as the nature of the microbe, which is species and strain specific [2, 72]. This is supported by one of our prior investigations in which the FAME profile of the oleaginous yeast *C. tropicalis* ASY2 cultured on SWW [19] was completely different from that of *A. caespitosus* ASEF14 used in the current investigation by its content, quantity, and fermentation days. For example, methyl oleate was major fatty acid found in yeast, but in fungi, methyl stearate was the dominant fatty acid. Table 3 shows the differences in the FAME profiles of different *Aspergillus* species when grown on different agro-industrial wastes. *A. niger* 364 and LFMB1 strains cultivated on waste cooking oil had high methyl oleate levels of 88.8 and 69%, respectively. Kakkad et al. [6] found that growing *A. candidus* in two different agro wastes, namely, sugarcane bagasse and banana peel, yielded 36.85% and 19% methyl linoleate, respectively. Methyl palmitate (24.95%) and methyl linolenate (42.66%) were abundant in *A. niger* grown on sugarcane effluent [73]. Generally, fungal FAME profiles are dominated by C16 and C18 carbon series, which are important fatty acids for biodiesel generation. This composition is quite similar to the often used biodiesel feedstock, i.e., vegetable oils.

**Table 3:** Comparison of FAME profile of *A. caespitosus* ASEF14 with different *Aspergillus* spp. and vegetable oils used in biodiesel production

S. No	Culture	Substrate/ waste type	Fatty acid profile								References
			C14:0	C16:0	C16:1	C18:0	C18:1	C18:2	C18:3	C20:0	
1	*A. caespitosus* ASEF14	SM	2.6	–	–	32.22	17.29	19.12	9.09	–	Present study
		SWW	–	0.95	0.32	64.77	14.11	12.81	–	0.02	
2	*A. oryzae*	Potato processing wastewater	4	11.6	15.6	19.3	30.3	6.5	5.5	2	[13]
3	*A. niger*	Sugarcane distillery wastewater	–	24.94	–	5.25	17.23	42.66	9.92	–	[73]
4	*A. terreus* IBB M1	Copra cake	14	16.27	0.18	7.72	16.57	19.97	1.11	0.72	[88]
5	*A. candidus* IBB G4	Sugarcane bagasse	–	0.4	–	0.5	14.4	36.8	–	–	[6]
		Banana peel	–	21.6	–	22.6	25	19	–	–	
6	*A. candidus*	Whey waste	–	21.50	–	8.7	31.50	38.3	–	–	
7	*A. niger* 364	Waste cooking olive oil	–	8.9	–	2.7	80.8	6.9	–	0.7	[89]
8	*A. niger* LFMB1		–	8.5		2.4	69	8	–	12.1	
Commercial feedstock											
9	Soybean oil		–	10–12	–	3–5	18–26	49–57	6–9	–	[79]
10	Rapeseed oil		–	2–6	–	4–6	52–65	18–25	10–11	–	[90]

Thus, the FAME yields obtained from ASEF14 on untreated, cheap, and renewable substrates such as SWW demonstrated the potential of the fungus in one-step saccharification-oil accumulation, allowing the direct transformation of oil-rich biomass into biodiesel. Owing to its rich saturated (C18:0; C20:0) and monounsaturated FAMEs (C18:1), as well as the absence of methyl esters with P4 double bonds, the produced fungal oil is expected to have favorable fuel qualities, such as high cetane number and enhanced oxidative stability. All these characteristics imply that the oleaginous fungi *A. caespitosus* ASEF14 is a promising candidate for microbial biodiesel production.

### Evaluation of fuel properties of *A. caespitosus* ASEF14 grown on SM and SWW

Direct assessment of biodiesel fuel qualities is difficult, expensive, and time-consuming, requiring a large amount of fuel sample. As a result, mathematical equations and prediction models have been developed to predict biodiesel properties from FAME composition [74]. These equations were previously reported for *A. terreus* IBB M1 [75], *A. candidus* IBB G4 [6], and *Yarrowia lipolytica* [76]. For the commercial sale of fungus-derived fuels, it must meet the criteria set by international standards ASTM 6751-3 (USA), EN 14214 (Europe), and the Bureau of Indian Standard (IS 15607-05) [77]. On day 6, FAMEs derived from *A. caespitosus* ASEF14 grown on SM and SWW exhibited desirable FAME profiles; it was expected to have good fuel properties signifying their potential feasibility for biodiesel and are presented in Table 4. When grown on SM, the saturated, monounsaturated, and polyunsaturated FAMEs were 53.9, 16.23, and 4.82%, respectively. Similarly, the fungal FAMEs of SWW were rich in saturated methyl esters (43.82%), MUFAs (9.62%), and PUFAs (10.87%) and composed primarily of methyl stearate (C18:0, > 40%) and palmitate (C16:0, 20%) as major saturated FAMEs. The density of fungal biodiesel from SM and SWW was 0.65 and 0.56 g cm^–3^, which is slightly lower than standard limits (0.86–0.90 g cm^–3^). The KV, IV, CP, and PP were in accordance with all biodiesel standards indicating the potential use of fungal oil as biodiesel. IV is a crude measure of the degree of unsaturation of biodiesel and is often used in connection with its oxidative stability. SV indicates the number of TAGs present in total lipid, and HHV depends on both IV and SV. Therefore, we calculated both SV and IV empirically from the fatty ester composition of in situ transesterified lipids and were found to be in good agreement with each other. The IV of fungal oil was low in SM (27.85 g I_2_/100 g of oil) and SWW (30.52 g I_2_/100 g of oil), which are below the EN specification (120 max), indicating good oxidative stability of the oil [78]. The SV values were 142.10 and 127.25, whereas HHV values were 29.8 and 25.56 MJ kg^–1^ for SM and SWW, respectively. OS is an important yardstick to determine the self-life of the engine. In this study, the OS value of fungal biodiesel derived from SM and SWW were 27.06 and 13.44 h, respectively. This high OS content might be due to more number of double bonds or unsaturated bonds in the oil.

**Table 4:** Fuel properties of FAME produced from *A. caespitosus* ASEF14 grown on SM and SWW

Fuel quality parameters	Fungal oil from SM	Fungal oil from SWW	Approved limits of biodiesel standard		
			US ASTM D6751	Europe EN 14214	India IS 15607
Saturated fatty acid (%)**	53.46	43.82	–	–	–
Monounsaturated fatty acid (%)**	16.23	9.62	–	–	–
Polyunsaturated fatty acid (%)**	4.82	10.87	–	–	–
Degree of unsaturation**	25.87	31.36	–	–	–
Density (g cm^–3^)*	0.65	0.56	0.86–0.90	0.86–0.90	0.86–0.90
Kinematic viscosity (mm^2^ s^–1^)*	3.32	2.62	1.9–6.0	3.5–5	2.5–6.0
Iodine value (mg I_2_100 g^–1^) *	27.85	30.52	–	120 max	–
Saponification value (mg g^–1^)*	142.10	127.25	0.50 max	0.50 min	0.5
Cloud point (°C)*	– 3.59	– 4.66	– 15 to – 5	–	–
Pour point (°C)*	– 10.73	– 11.90	– 20 to – 6	–	–
Higher Heating Value (MJ kg^–1^)*	29.80	25.56	NS	NS	NS
Oxidation stability (h) *	27.06	13.44	3 h min	6 h min	–
Cetane number*	78.44	82.32	47 min	51 min	51 min
Concentration of γ-linolenic acid (C18:3) (%)**	< 5	< 9	NS	12 max	NS
FAME having ≥ 4 double bonds **	ND	ND	NS	1 max	NS

^*^predicted values as mentioned in Methods

^**^experimentally determined values by GC-FID according to standard AOAC methods

*ND* Not detected, *NS* not specified

Cetane number (CN) is a property of fuel that decides its ignition quality. The methyl esters of stearic acid (C18:0), which are of relevance to biodiesel, have been found to possess the highest CN (> 80) [79]. We found that the methyl esters of long-chain saturated fatty acids, namely stearic acid (C18:0) and palmitic acid (C16:0), were present in a higher percentage in the FAME profile of *A. caespitosus* ASEF14 grown on SWW. The CN values for fungal biodiesel of SM and SWW were 78.44 and 82.32, respectively, which are substantially higher than conventional petro-diesel fuels that fall within the range of 47 to 51. Previously, the yeast *Candida tropicalis* ASY2 biodiesel had a CN value of 61 [14]. The CN value of 70 was reported earlier for biodiesel from microalga *Spirulina platensis* [80]. Higher CN in the fungus-derived biodiesel can be attributed to an increase in saturated FAME content and its chain length. Higher CN results in higher combustion efficiency, improved engine performance, and cleaner emissions so that it can be recommended for use in the high-speed engine (above 800 rpm) [80]. Conversely, a fuel with low CN will cause difficulty in engine starting, generating higher noise and exhaust smoke [80]. Another crucial fuel quality that has been included in EN 14214 specification is the concentration of linolenic acid (C18:3), produced below the specified limit of 12 max, and fatty esters with ≥ 4 double bonds that were not detected in both SM and SWW of fungal FAMEs (Table 4). These values are in the acceptable range of international biodiesel standard norms, suggesting the suitability of SWW grown ASEF14-derived biodiesel.

### Integrated decontamination of SWW and lipid production by *A. caespitosus* ASEF14

The fungal strain ASEF14 was grown on SWW for ten days. After fermentation, the mycelium was harvested, and lipids were extracted using chloroform and methanol. The spent wastewater was filtered and used to analyze the physicochemical parameters and then compared with raw SWW (Table 5). Physicochemical analysis of raw and treated SWW showed that the fungal strain increased the pH from acidity (pH 6) to alkalinity (pH 9.13) due to the secretion of amines and related compounds [81]. The electrical conductivity was very high in SWW, nearly 6.2 dS m^–1^; it was reduced to 4.1 dS m^–1^ after the treatment. The organic substances such as TS, TDS, and TSS from tapioca tuber extraction in the wastewater were reduced to 58.6%, 53%, and 35.2%, respectively. The nutrients present in the wastewater, such as total nitrogen (0.44 g L^–1^) and phosphate levels (1.06 g L^–1^) (Table 5), were removed to a level of 89.3% and 94.5%, respectively. This was comparable with our earlier reports on decontamination of SWW by oleaginous yeast *C. tropicalis* ASY2 that registered removal of nitrate (NO_3_), ammonia (NH_4_), and phosphate (PO_4_) ions in the level of 100%, 98%, and 85%, respectively [14]. In our study, the initial starch content of 6.1 g L^–1^ was in accordance with previous reports [82, 83], and it was adjusted to 30 g L^–1^ before the fermentation process started. At the end of fermentation, the residual sugar level in the SWW was 11.12 g L^–1^ indicated the starch utilization efficiency of fungi. The dissolved oxygen (91.3%), BOD (74%), and COD (47%) were reduced to a considerable level. The change in the COD and BOD could be attributed to the effective use of nutrients available in wastewater by the fungi. The nutrient removal efficiency of *A. caespitosus* ASEF14 was compared with the following studies. Xue et al. [12] used monosodium glutamate wastewater as a fermentable medium for lipid production using *Rhodotorula glutinis*. Because the wastewater contained nitrogen, favoring biomass growth but not lipid accumulation, glucose was added at various levels, namely initial, fed-batch, and feedback addition. Of these, the feedback addition yielded 20% of lipid content and 45% COD removal. Xue et al. [58] cultivated *R. glutinis* by non-aseptic fermentation using corn starch wastewater for lipid production and achieved 35% of lipid content and 80% of COD reduction. Muniraj et al. [17] treated PPW, which efficiently removed soluble COD, total soluble nitrogen, and total soluble phosphorus up to 60% and 90%, 100% and 98%, and 92% and 81%, and produced lipid yield of 2.8 and 3.6 g L^−1^ by *A. flavus* I16–3 and *M. rouxii,* respectively. Nzayisenga et al. [84] treated municipal wastewater using four algal strains (*Chlorella vulgaris, Desmodesmus* sp.*, Ettlia pseudoalveolaris,* and *Scenedesmus obliquus*) along with lipid production. All strains removed more than 75% of the total nitrogen and phosphorus content of the treated wastewater after 8 days.

**Table 5:** Physicochemical characteristics of treated SWW

Parameters	Values (g L^–1^)		% reduction
	Raw SWW	Treated SWW	
pH	4.30 (± 0.0)	9.13 (± 0.0)	–
Electrical conductivity (dS m^–1^)	6.21 (± 0.0)	4.11 (± 0.0)	–
Salinity (ppt)	4.53 (± 0.0)	3.15 (± 0.0)	30.6
Total dissolved solids (TDS)	4.07 (± 0.0)	2.64 (± 0.1)	35.2
Total suspended solids (TSS)	6.67 (± 0.8)	3.14 (± 0.5)	53.0
Total solids (TS)	6.90 (± 0.1)	2.86 (± 0.6)	58.6
Total phosphate (TP)	1.06 (± 0.0)	0.06 (± 0.0)	94.5
Total nitrogen (TN)	0.44 (± 0.0)	0.05 (± 0.0)	89.3
Dissolved oxygen (DO)	7.84 (± 0.6)	0.68 (± 0.0)	91.3
Biochemical oxygen demand (BOD)	2.56 (± 0.5)	0.67 (± 0.0)	74.0
Chemical oxygen demand (COD)	76.00 (± 6.9)	40.27 (± 0.5)	47.0
Starch	6.10 (± 0.8)	–	–
Starch*	30.00 (± 0.0)	11.12 (± 1.7)	62.9
Cyanide (mg L^–1^)	5.2 (± 1.0)	2.4 (± 0.02)	53.84

Values represent mean (± standard error) (n = 3)

^*^ The initial starch concentration in the SWW was adjusted to 30 g L^–1^

Similarly, oleaginous fungus *A. caespitosus* ASEF14 effectively used various nutrients in the wastewater as growth components and produced high lipid content (37.27%). In addition, the fungal strain reduced cyanide toxicity levels by up to 50% which was less than from our previous report [14] indicates the tolerable level of fungi to the toxicant cyanide.

## Conclusion

Our study clearly indicates that, there are marked differences between oleaginous yeast and fungi in terms of substrate utilization efficiency, sugar requirement, lipid accumulation pathways, FAME composition, and respective fuel properties when cultivated on the same substrate. Both organisms have their own advantages and disadvantages. *A. caespitosus* ASEF14 exhibited high lipid content of 54% in SM and 37.27% in SWW with a fatty acid profile similar to that of vegetable oils (soybean and rapeseed), which are currently employed in biodiesel production. Moreover, SWW-derived biodiesel properties (CN: 82.32, IV: 30.53 g g^–1^, KV: 2.62 mm^2^ s^–1^, CP: 4.66 °C, and PP: 11.90 °C) were in accordance with the existing biodiesel standards. Therefore, the use of oleaginous fungus *A. caespitosus* ASEF14 isolated from SWW as myco-diesel holds immense potential for current diesel substitution. In addition, it is an efficient method to decontaminate SWW by reducing COD, BOD, various nutrients, and toxicants at a considerable level. This kind of myco-diesel or biodiesel has the potential to be used as a substitute for fossil fuels, especially when several countries are attempting to find alternative sources, owing to its low cost and eco-friendly nature of production.

## Data Availability

All data generated or analyzed during this study are included in this published article.

## Abbreviations

SWW: Sago processing wastewater
SM: Synthetic medium
COD: Chemical oxygen demand
BOD: Biological/Biochemical oxygen demand
ASTM: American Society for Testing and Materials
EN: European standards
DCW: Dry cell weight
ANOVA: Analysis of variance
KV: Kinematic viscosity
SV: Saponification value
IV: Iodine value
HHV: Higher heating value
CV: Cetane number
LCSF: Long-chain saturated factor
CFPP: Cold filter plugging point
CP: Cloud point
PP: Pour point
DU: Degree of unsaturation
